# Functional connectivity response to acute pain assessed by fNIRS is associated with BDNF genotype in fibromyalgia: an exploratory study

**DOI:** 10.1038/s41598-022-23476-3

**Published:** 2022-11-06

**Authors:** Álvaro de Oliveira Franco, Guilherme de Oliveira Venturini, Camila Fernanda da Silveira Alves, Rael Lopes Alves, Paul Vicuña, Leticia Ramalho, Rafaela Tomedi, Samara Machado Bruck, Iraci L. S. Torres, Felipe Fregni, Wolnei Caumo

**Affiliations:** 1 Laboratory of Pain and Neuromodulation, Hospital de Clínicas de Porto Alegre (HCPA), 2400 Rua Ramiro Barcelos, Porto Alegre, RS 90035-003 Brazil; 2 Post-Graduate Program in Medical Sciences, School of Medicine, Universidade Federal Do Rio Grande Do Sul (UFRGS), Porto Alegre, Brazil; 3 Center of Pain Pharmacology and Neuromodulation: Pre-Clinical Research LAFDOR, Centro de Pesquisa Experimental, HCPA, Porto Alegre, Brazil; 4 Laboratory of Neuromodulation and Center for Clinical Research Learning, Physics and Rehabilitation Department, Spaulding Rehabilitation Hospital, Boston, MA USA; 5 Pain and Palliative Care Service, HCPA, Porto Alegre, Brazil; 6 Department of Surgery, School of Medicine, UFRGS, Porto Alegre, Brazil

**Keywords:** Predictive markers, Fibromyalgia, Chronic pain, Genetics of the nervous system, Neural circuits

## Abstract

Fibromyalgia is a heterogenous primary pain syndrome whose severity has been associated with descending pain modulatory system (DPMS) function and functional connectivity (FC) between pain processing areas. The brain-derived neurotrophic factor (BDNF) Val66Met single nucleotide polymorphism has been linked to vulnerability to chronic pain. In this cross-sectional imaging genetics study, we investigated fibromyalgia, the relationship between BDNF Val66Met heterozygous genotypes (Val/Met), and the functional connectivity (FC) response pattern to acute pain stimulus in the motor (MC) and prefrontal (PFC) cortex assessed by near-infrared spectroscopy (fNIRS) before and after a cold pressor test utilizing water (0–1 °C). Also, we assessed the relationship between this genotype with the DPMS function and quality of life. We included 42 women (Val/Val = 30; Val/Met = 12) with fibromyalgia, ages 18–65. The MANCOVA comparing Val/Met to Val/Val genotypes showed higher ΔFC between left(l)-PFC—l-MC (β = 0.357, *p* = 0.048), l-PFC—right(r)-PFC (β = 0.249, *p* = 0.012), l-PFC—r-MC (β = 0.226, *p* = 0.022), and l-MC—r-PFC (β = 0.260, *p* = 0.016). Val/Met genotypes showed higher efficiency of the DPMS and lower disability due to pain. Here we show that fibromyalgia patients carrying the Val/Met BDNF genotype presented an increased ΔFC across MC and PFC in response to acute pain associated with differences in acute pain perception and fibromyalgia symptoms.

## Introduction

Fibromyalgia (FM) is a chronic primary pain syndrome defined by widespread pain^1^ associated with fatigue, diffuse tenderness, hyperalgesia, sleep disorders, cognitive and mood difficulties, as well as psychological and psychiatric disorders^2^. It is a condition more prevalent in women than men^3^, affecting from 0.4 to 9.3% of the population worldwide^4^. Additionally, central sensitization (CS) seems to play a major role in FM pathology in as much as the pain hypersensitivity likely results from central nervous system (CNS) amplification of painful states due to higher pain facilitation over inhibition^2,5^. These central mechanisms related to CS may also explain the occurrence of cognitive, behavioral, and emotional symptoms in FM and its significant coexistence with mental illness^2,5^. Nevertheless, establishing objective biomarkers for FM remains an elusive task.

Many attempts to tackle the heterogeneity and non-specificity of FM manifestations have been made by describing this complex disease in terms of its subgroups, which vary in type and degree of physical and cognitive-affective symptoms^6^. The descending pain modulatory system (DPMS) facilitates or inhibits pain, comprising cortical and subcortical CNS regions such as the ventrolateral periaqueductal grey, rostroventral medulla, and anterior cingulate cortex, among other regions, with significant involvement of the prefrontal cortex (PFC) and motor cortex (MC)^7,8^. We previously reported that the dysfunction of the DPMS in women with FM was associated with higher functional connectivity (FC) between the left MC and bilateral PFC assessed by functional near-infrared spectroscopy (fNIRS)^9^. Higher severity of FM symptoms has been associated with dysfunction of the DPMS^10^, the latter assessed by a standardized quantitative psychophysical protocol known as the conditioned pain modulation (CPM) test^7,10^.

The relationship between PFC and MC with endogenous pain modulation remains an active area of research, with conflicting evidence for structure, functional, and organization changes in the primary motor cortex in chronic pain^11^. Both the PFC and the MC are therapeutic targets for non-invasive brain stimulation (NIBS), apparently resulting in the top-down modulation of the respectively emotional^12^ and sensory-discriminative components of pain perception^11–14^. PFC dysfunction in chronic pain is associated with changes in neurotransmitters, gene expression, and neuroinflammation and with alterations in structure, activity, and connectivity during acute and chronic pain^15^. Also, left PFC activation assessed by fNIRS after thermal stimuli seem to be a sensitive marker of FM patients with more severe clinical symptoms^16^.

Neuroplasticity is a fundamental biological process for CS, while the brain-derived neurotrophic factor (*BDNF*) is a candidate gene with a critical impact in regulating synaptic plasticity in human brains^5,17^. A single nucleotide polymorphism (SNP) in the *BDNF* gene (c.196G > A, dbSNP: rs6265) causes a valine to methionine substitution at amino acid residue 66 in the *BDNF* prodomain, which has been correlated to reduced activity-dependent *BDNF*-secretion^18^. The Val66Met polymorphism seems to moderate the relationship between stress and depression^19^, whereas Val66Met carriers with primary dysmenorrhea exhibited a diverse FC expression within the DPMS assessed by functional magnetic resonance imaging^20^. Finally, increased BDNF serum concentration in FM has been shown to positively correlate with a lower pain pressure threshold^21^ and the dysfunction of the DPMS^10^. Therefore, the *BDNF* may be an intermediate between the DPMS dysfunction and the emergent phenotypes characterized by CS and chronic pain, possibly underlying the functional pattern between cortical areas for pain processing and perception.

Regarding the fNIRS, it is particularly suitable for investigating cortical activation and assessing the hemodynamic response related to the coupling between transient neuronal activation and subsequent cerebral blood flow. Consequently, it is adequate for exploring the changes in functional cortical patterns associated with chronic and acute pain. It is not as expensive as fMRI, being amenable and versatile to interactive paradigms and presenting a better temporal resolution than fMRI^22,23^.

Based on the facts mentioned above, it is reasonable to test the hypothesis that FM patients with different genotypes of the *BDNF* Val66Met polymorphism present distinct cortical processing in response to acute pain indexed by changes in functional connectivity (ΔFC) parameters and that this is associated with DPMS efficiency and quality of life due to pain. For this reason, this imaging genetics and exploratory study assessed the relationship between *BDNF* Val66Met genotypes and change in response to a cold pressor test in FC set by fNIRS in the MC and PFC cortex (primary outcome). Also, we evaluated the relationship between the *BDNF* Val66Met genotype with the DPMS and the impact of symptoms on quality of life due to fibromyalgia (secondary outcomes).

## Materials and methods

### Procedure and participants

The Institutional Review Board approved this cross-sectional study under the number 2017–0329 of the Hospital de Clínicas de Porto Alegre (HCPA), Brazil, and registered in the Certificate of Presentation of Ethical Appreciation (CAAE registry No. 72793617.4.0000.5327), according to the Declaration of Helsinki. The protocol was developed following the Strengthening Reporting of Observational Studies in Epidemiology Checklist (STROBE). All participants provided written informed consent before their inclusion. The study enrollment period ranged from January 2018 to January 2021.

### Participant recruitment

A convenient sample of forty-two females aged 18–65 years, right-handed, and diagnosed with FM were enrolled from the outpatients' chronic pain wards of the HCPA, Porto Alegre, Brazil, and also after digital media divulgation. FM was defined according to the 2016 diagnosis criteria of the American College of Rheumatology (ACR)^24^. The ACR score comprises two scales: the widespread pain index (WPI) and symptom severity score (SSS). Criteria were defined as: (i) WPI ≥ 7 and SSS ≥ 5 OR WPI 4–6 and SSS ≥ 9. (ii) Generalized pain: pain in 4/5 regions. (iii) Presence of symptoms ≥ three months. (iv) The FM diagnosis is irrespective of other conditions. ACR score evaluation was applied by physicians with more than ten years of experience in pain care. Patients had to present active clinically significant symptoms, so they should present daily disability for the routine activities due to FM during the three months preceding the enrollment. They needed to report a score of at least 50 mm on the 0–100 mm visual analog scale (VAS 0–100 mm) for the average day in the last three months. All examiners and patients were fluent in Portuguese. Only female patients were included due to the significantly higher prevalence of FM in women than in men^3^.

Subjects were excluded if they presented a positive history of rheumatoid arthritis, lupus, autoimmune disease, neurological or oncological disease, uncompensated clinical disease (e.g., ischemic heart disease, chronic kidney disease, and hepatic disease.). They were also excluded if they had used cannabis or recreational psychotropic drugs in the last six months.

### Instruments and assessment outcomes

#### Dependent and independent variables

The dependent variables were the difference in ΔFC between the ipsi- and contralateral MC and PFC after and before a cold pressor test (CPT) assessed by fNIRS. Secondary outcomes included the efficiency of the DPMS—assessed by change on the numerical pain scale (NPS) during the conditioned pain modulation (CPM) test—and disability due to FM symptoms—evaluated by the fibromyalgia impact questionnaire (FIQ). The main interest independent variables were the BDNF polymorphisms. Covariates included the intensity of chronic pain, the BDNF serum levels, pain catastrophizing, depressive symptoms, sleep quality, sociodemographic characteristics, clinical and psychiatric chronic diseases, and psychotropic and analgesic medications.

#### fNIRS acquisition

**Figure 1:**
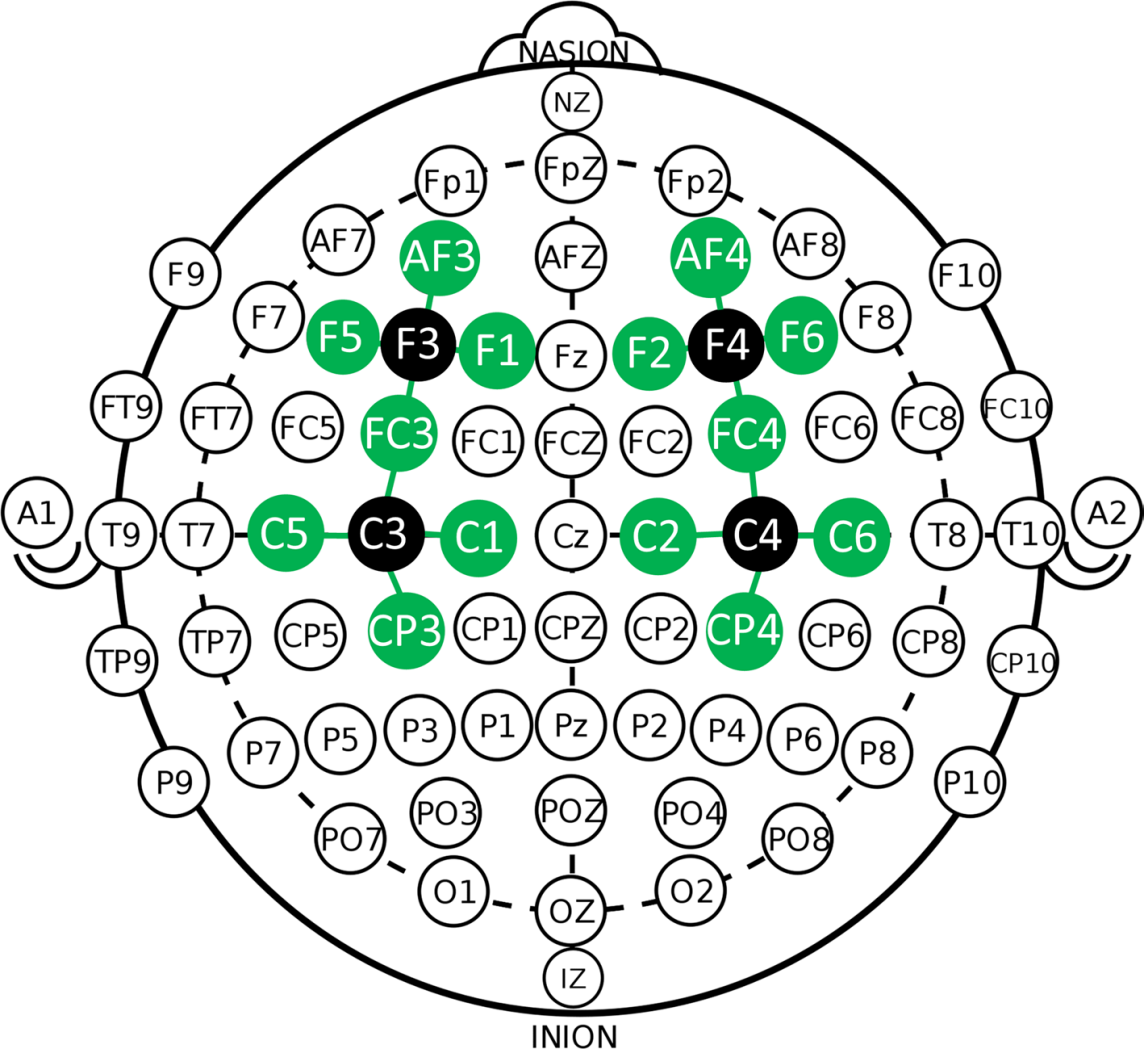
fNIRS montage. *fNIRS* functional near-infrared spectroscopy.

Functional connectivity was evaluated by fNIRS, with a NIRx^®^ continuous waveform NirScout^®^ near-infrared spectroscopy device (NIRx Medical Technologies, Glen Head, NY, USA), with a scan rate of 15 Hz, dual-wavelength light-emitting diode sources (760 and 850 nm). Four sources and 14 detectors were spaced about 3 cm apart and placed over the scalp. The caps were bought from EASYCAP^®^. The montage was intended to create 16 channels and cover the bilateral dorsal prefrontal cortex as well as the bilateral MC. The international 10–10 electroencephalography system was employed to guide probe positioning (Fig. 1). In our montage, we placed sources (S) in the F3, F4, C3, and C4 locations and detectors (D) in the AF3, F5, FC3, F1, C5, CP3, C1, AF4, F6, FC4, F2, C6, CP4, and C2 locations.

We defined four ROIs: left (l-) PFC (S: F3; D: AF3, F1, FC3, F5), l-MC (S: C3; D: FC3, C1, CP3, C5), right (r-) PFC (S: F4; D: AF4, F6, F2, FC4), r-MC (S: C4; D: FC4, C6, CP4, C2). Software equipment was NIRStar^®^ version 14.2 (NIRx Medical Technologies, Glen Head, NY, USA). The acquisition consisted of one session of admeasurement and fNIRS acquisition, starting with 7 min of resting state, followed by a CPT (water at 0–1 °C) and then another 7 min of resting state.

A black cover was placed over the adjusted NIRS cap on the scalp to reduce environmental light disturbance. Recordings were resumed only if source-detection calibration and recording-checked signal quality retrieved an “excellent” quality in at least 14 out of 16 channels, with tolerance for the other two channels to be at least "acceptable". A qualitative scale gauged signal quality based on gain, amplitude, coefficient of variation of noise, and dark noise. For fNIRS recording, subjects sat on a comfortable armchair and maintained a still position. They fixated their gaze on a black cross fixed on the front wall at eye level 1.5 m ahead of the armchair, and they were asked to try to think of nothing.

#### fNIRS and cold pressor test

**Figure 2:**
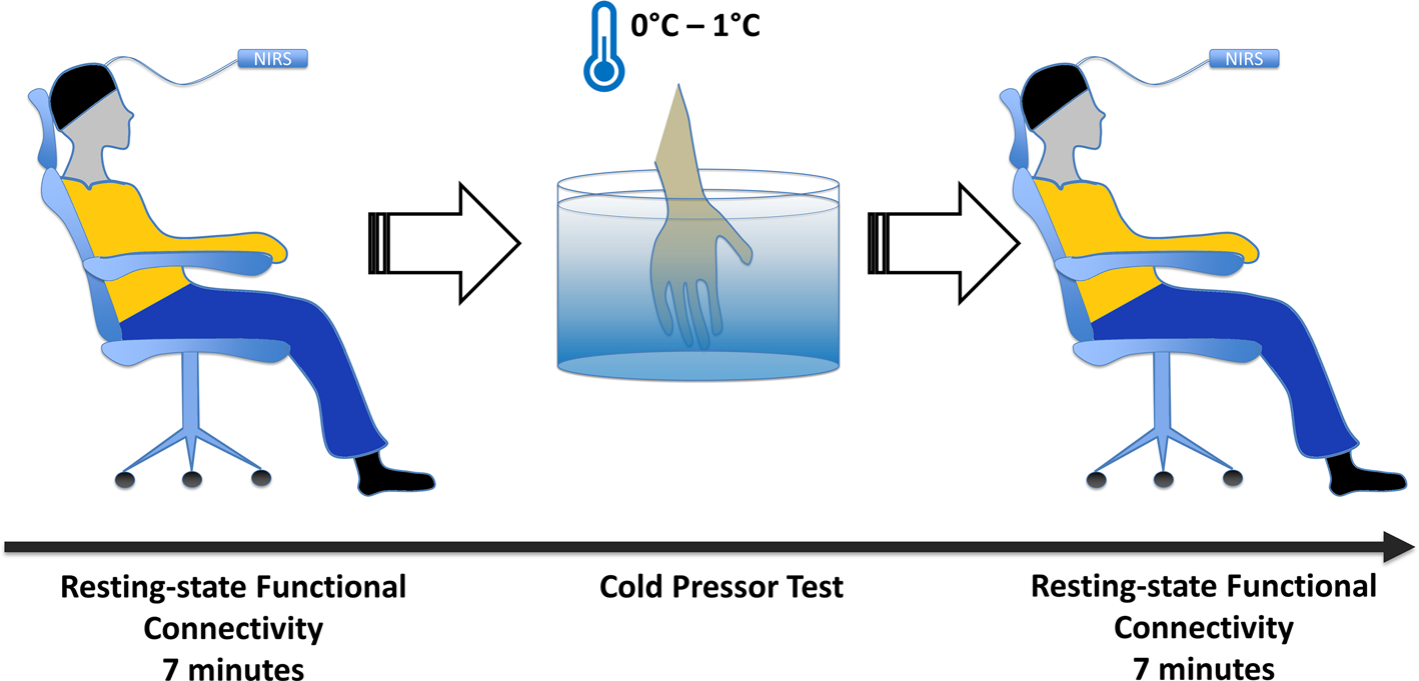
fNIRS connectivity assessment. The cold pressure test lasted from a minimum of 10 s until maximum tolerance to pain. Pre and post-stimuli functional connectivity yielded a difference in Z values (ΔFC).

The signal was recorded for 7 min in a resting state. The CPT was performed while recording the cortical activation. CPT consisted of right-hand immersion up to the wrist in cold water (0–1 °C, measured by a digital thermometer) for at least 10 s and until the maximum tolerated pain^25,26^. After they took out the hand, we measured the resting-state cortical activation for seven more minutes—with the right-hand volar aspect laying on a towel previously put on the subjects’ lap (Fig. 2).

#### Preprocessing and functional connectivity analysis

Raw data was analyzed through the Brain AnalyzIR^®^ toolbox^27^ in the MATLAB environment (MathWorks, Natick, MA, USA). Raw data were downsampled to 1 Hz to adequately address the high level of temporal autocorrelation in fNIRS signals, then converted to optical density into relative oxyhemoglobin concentration variations (HbO) through the modified Beer-Lambert Law^28,29^. We have utilized the oxyhemoglobin signal in the analysis due to evidence that this variable is the most sensitive to inferring hemodynamic response based on neurovascular coupling^30–32^.

Structured noise (e.g., physiological noise) was corrected through an autoregressive pre-whitening model, whereas motion artifacts were treated through a robust regression using iterative reweighting. Both corrections used no band/high/low filtering^33,34^. This method was shown to effectively address serially correlated errors, colored noise, and motion artifacts^33,35^. All possible pairs of channels across the time series were correlated, resulting in Pearson correlation values that underwent a Fisher Z-transformation. The difference in ROI-ROI FC (ΔFC) for each patient was retrieved by subtracting the mean ROI-ROI Z-value before CPT from the mean ROI-ROI Z-value after CPT. The ΔFC was submitted to ROI-ROI group analysis (6 ROI-ROI pairs × 42 subjects)^36^.

#### Secondary outcomes

- DPMS function was assessed through a CPM test—expressed by the difference in quantitative sensory testing (QST) before and concurrently to a CPT serving as a heterotopic nociceptive stimulus. First, a thermode was on the ventral non-dominant forearm to define the average of three temperatures (T0) corresponding to a patient’s QST report score of 6/10 (Numerical Pain Scale (NPS) 0–10). Second, after five minutes, subjects were asked to immerse their dominant hand up to their wrist into the water at a temperature of 0–1 °C for 15 s. QST was reassessed with a pain score NPS(T1) in relation to the evoked pain by the thermode applying the T0 temperature in the ventral non-dominant forearm (QST + CPM-test). Third, the CPM-test score was calculated by the difference in the pain score on NPS(T1) and the NPS(T0). Negative values in the CPM score represent a proper DPMS function, whereas CPM scores ≥ 0 represent an impaired function.
- *Fibromyalgia Impact Questionnaire (FIQ)*, adapted for use in Brazil^37^, was used to evaluate the impact of symptoms on quality of life. It comprises ten domains and items with higher scores indicating higher disability due to pain in performing routine daily living activities, including the presence of fatigue, morning stiffness, and mood and psychiatric symptoms.

#### Sociodemographic, clinical, and psychological measures

Demographic data, medical comorbidities, medications use, and daily doses were evaluated using a standardized questionnaire. Psychiatric diagnoses were assessed by the Mini-international Neuropsychiatric Interview (MINI)^38^. Depressive symptoms were assessed by the Beck Depression Inventory—Second Edition (BDI-II)^39^. All questionnaires were applied using the validated Portuguese version.

*Central Sensitization Inventory* (CSI) was used to assess the severity of symptoms related to CS^40^.

*Pittsburgh Sleep Quality Index* (PSQI) evaluated sleep quality and disturbances over the last month^41^.

*The Brazilian Portuguese Pain Catastrophizing Scale* (BP-PCS) was used to evaluate pain catastrophizing^42^.

*Pain Visual Analogue Scale* (VAS) was used to assess pain intensity on most days of the last three months ranging from zero to 100 mm (i.e., worst possible pain).

#### Serum BDNF and BDNF Genotype assessments

The BDNF serum levels were assessed by enzyme-linked immunosorbent assay (ELISA) monoclonal antibodies specific for BDNF (R&D Systems, MN, United States, ChemiKine BDNF Sandwich ELISA kit, CYT306, Chemicon/Millipore, Billerica, MA, USA), and the Enzyme-linked Immunosorbent. Essays were performed in duplicates to assess intra-assay variation. Inter-assay variation was addressed using two plates per kit over two different days within the same week. All protocols followed the manufacturer’s instructions. The lower detection limit for BDNF was 7.8 pg/ml. ELISA was measured by optical density with a wavelength of 450 nm (GloMax^®^-Multi Microplate Reader; Promega, WI, USA). Multiplexing assay measurements were conducted in the Bio-Plex^®^-200 instrument (Bio-Rad). Total protein was assessed using bovine serum albumin following the Bradford method.

**Figure 3:**
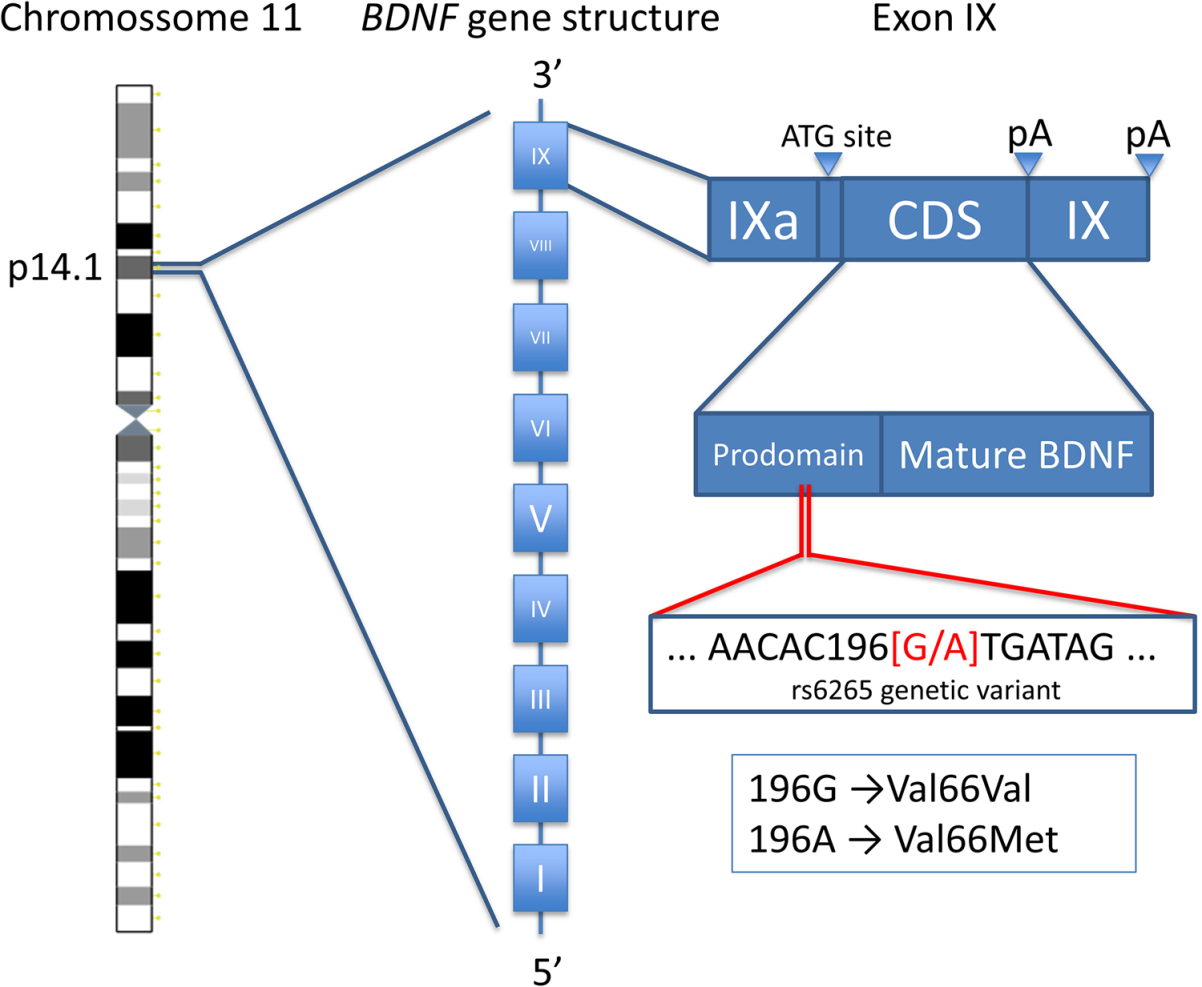
Representation of the *BNDF* location and structure. The Val66Met (rs6265) polymorphism of the *BDNF* gene consists of a substitution of valine for methionine in the *BDNF* prodomain. *BDNF* exons are represented by boxes noted with roman algorisms, while lines represent introns. The SNP locus is in the prodomain region of the IX exon. *CDS* coding DNA sequence. *pA* polyadenylation sites. *ATG site* initiation codon. *BDNF* brain-derived neurotrophic factor; *Val/Val* Val66Val homozygous. *Val/Met* Val66Met heterozygous.

Blood samples were collected in 4 ml tubes with ethylenediamine tetraacetic acid. Total DNA was extracted and purified using PureLink^®^ Genomic DNA Kit (Invitrogen, ThermoFisher Scientific). A StepOnePlusTM Real-Time PCR System (Applied Biosystems Inc, Foster City, USA) was used for genotyping the Val66Met *BDNF* (rs6265) polymorphism. A predesigned TaqManTM SNP genotyping assay was employed (Thermo Fisher Scientific; catalog 4,351,379, assay ID_C1159275810). A representation of the structure of the human *BDNF* gene and its location on chromosome 11 can be seen in Fig. 3.

### Statistical analysis

Assessment of data distribution was performed using the Shapiro–Wilk test. Descriptive statistics were used to summarize the main characteristics of the sample. T-tests for independent samples were used to compare continuous variables between groups. Descriptive statistics were used to summarize the main socio-demographic features of the sample. The chi-squared and Fisher's exact tests were used to compare groups for categorical variables. Spearman's correlation was used to analyze the relationship between the six ROI-ROI ΔFC Z-values, serum BDNF, ACR-score, BDI-II, BP-PCS, and FIQ.

A multivariate covariance analysis (MANCOVA) model was used to explore the relationship between the ROI − ROI ΔFC Z-values according to the BDNF genotype. Independent linear regression analysis models using the best subset selection was used to identify the possible clinical severity symptoms and serum BDNF associated with the dependent variables (ROI-ROI ΔFC Z-values) independently. Bonferroni's correction was performed for multiple comparisons.

A generalized linear mixed model (GLMM) was used to compare the change in NPS (0–10) during the CPM-test and the FIQ scores between groups of Val homozygous for *BDNF* (Val/Val) and heterozygous for the Met allele (Val/Met), followed by Bonferroni's Multiple Comparison Test. The model was adjusted to ACR score and serum BDNF. All analyses were performed with two-tailed tests. We accepted a type I error of 5%. For all analyses, we considered a two-tailed type I error α = 0.05. Statistical analysis was performed by the IBM SPSS Statistics for Windows, version 22.0 (IBM Corp., Armonk, N.Y., USA).

## Results

### Patient characteristics

The sample was composed of 42 female subjects, 30 were homozygous for the *BDNF* (66)Val allele (Val/Val), and 12 were heterozygous for the Val66Met allele (Val/Met). The clinical and demographic characteristics are presented in Table 1. The groups differed regarding years of education, the WPI index, and the FIQ.

**Table 1:** Demographic and characteristics according to the *BDNF* genotype.

	*BDNF* Val/Val (n = 30)	*BDNF* Val/Met (n = 12)	*p*-value
Body mass index (kg/m^2^)	30.0 (5.05)	27.40 (5.745)	0.157
Age (years)	49.733 (8.28)	44.667 (8.049)	0.079
Education (years)	11.066 (4.017)	15.727 (3.289)	0.001
Employed (yes)	16 (53.3%)	11 (91.7%)	
Smoking (yes)	9 (30%)	2 (16.7%)	
Alcohol use (yes)	13 (43.3%)	5 (41.7%)	
Opioid analgesic medication in use (yes)	8 (26.7%)	0 (0%)	
Non-opioid analgesic medication in use (yes)	30 (100%)	10 (83.3%)	
Chronic disease (yes)	15 (50%)	8 (66.6%)	
Hypertension (yes)	9 (30%)	2 (16.7%)	
Type 2 Diabetes Mellitus (yes)	2 (6.7%)	0 (0%)	
Asthma (yes)	2 (6.7%)	4 (33.3%)	
History of major depression disorder (yes)	23 (76.7%)	8 (66.7%)	
Use psychiatric drugs (yes)**	17 (56.7%)	11 (91.7%)	
Selective serotonin reuptake inhibitor in use (yes)	7 (23.3%)	3 (25%)	
Tricyclic antidepressant (yes/no)	5 (16.7%)	1 (8.3%)	
Benzodiazepine	6 (20%)	2 (16.7%)	
Visual Analogue Scale (10 cm)	8.178 (1.498)	8.209 (1.048)	0.948
Brazilian Pain Catastrophizing Scale	35.267 (10.935)	32.167 (12.276)	0.427
Central Sensitization Inventory	64.133 (17.254)	60.833 (17.903)	0.583
Fibromyalgia Impact Questionnaire	71.975 (15.031)	59.586 (19.547)	0.039
Pittsburgh Sleep Quality Index	12.933 (3.40)	12.917 (4.07)	0.99
ACR score	23.87 (3.34)	21.92 (4.19)	0.12
(i) Widespread pain index	14.567 (2.622)	12.333 (3.312)	0.026
(ii) Generalized pain: pain in 4/5 regions	10.267 (1.721)	9.583 (1.73)	0.253
BDNF serum (ng/ml)	37.679 (25.089)	27.087 (11.576)	0.170

Data are presented as mean and standard deviation (SD) or frequency (%) (n = 42).

*BDNF* brain-derived neurotrophic factor*. Val/Val* Val66Val homozygous*. Val/Met* Val66Met heterozygous*. ACR score* American College of Rheumatology score for fibromyalgia diagnosis.

### Univariate analysis

#### Comparisons of mean according to BDNF genotype on primary and secondary outcomes

The mean (standard deviation) of primary and secondary outcomes according to the Val/Met genotype are presented in Table 2. In the univariate analysis, we did not find a difference statistically significant between genotypes related to ΔFC intra- and inter-hemispheric. The heterozygotes showed lower scores on the QIF compared to homozygotes.

**Table 2:** Primary and secondary outcomes t-test between *BDNF* genotype groups (n = 42).

*BDNF* Val/Val genotype (n = 30)	*BDNF* Val/Met genotype (n = 12)	*p*-value
Mean (SD)	Mean (SD)	
**Primary outcome—ROI-ROI ΔFC**		
**ΔFC l-PFC—l-MC**^**$**^		
− 0.079185732557955 (0.312291438384725)	0.071756194819515 (0.235193737916397)	0.140
**ΔFC l-PFC—r-PFC**^**$**^		
− 0.090395179866225 (0.286240405013819)	0.070308044593503 (0.237241679754818)	0.093
**ΔFC l-PFC—r-MC**^**$**^		
− 0.095883175866177 (0.299206968556765)	0.042919540038922 (0.207586756768148)	0.150
**ΔFC l-MC—r-PFC**^**$**^		
− 0.099790748977071 (0.306940454691258)	0.046170791613494 (0.294900024441640)	0.167
**ΔFC l-MC—r-MC**^**$**^		
− 0.083263924666726 (0.323896895126145)	0.042149028486911 (0.325663749592960)	0.264
**ΔFC r-PFC—r-MC**^**$**^		
− 0.080938736528707 (0.328997349351637)	− 0.006936448860856 (0.303214680167078)	0.505
**Secondary outcomes**		
Mean (SD) and median and interquartile interval (Q-25–75)	Mean (SD) and median and interquartile interval (Q-25–75)	
**Change on Numerical Pain Scale (0–10) during conditioned pain modulation **^**¥**^		
− 0.87 (2.03) − 1 (− 5; 4)	− 2 (1.90) − 1(1.90; 1)	0.10
**Fibromyalgia Impact Questionnaire**^**¥**^		
73.27 (13.53) 74.86 (22.67; 94.65)	60.54 (18.31) 63.59 (17.68; 92.59)	0.00

Data are presented as the mean (standard deviation) and/or median interquartile.

*BDNF* brain-derived neurotrophic factor*. Val/Val* Val66Val homozygous*. Val/Met* Val66Met heterozygous*. ROI* region of interest*. ΔFC* difference in functional connectivity*. l-* left*; r-* right*. PFC* prefrontal cortex. *MC* motor cortex*. SD* standard deviation.

^**$**^Compared by t-test for independent samples. ^**¥**^Mann–Whitney Test.

#### Analysis of the relationships between outcomes according to BDNF genotype

The Spearman correlation analysis was used to explore the relationship between the ROI-ROI ΔFC with the following covariates according to Val66Met genotypes: ACR score, PCS, BDI-II, FIQ, serum BDNF (ng/ml), and the CPM test score. These correlations are presented in Table 3. We found a positive and moderate correlation between serum BDNF in Val/Val genotype with ΔFC in l-PFC—l-MC and l-PFC—r-MC. In contrast, in Val/Met patients, the ACR score was moderately and positively correlated with the ΔFC in l-FC—l-MC, l-MC—r-FC, and r-FC—r-MC. The FIQ score positively correlated with the ΔFC in l-MC—r-MC.

**Table 3:** Correlation among the ∆FC between regions of interest before and after a cold pressor test and potential confounding factors according to the *BDNF* genotypes (n = 42).

BDNF Val/Val genotype (n = 30)											
	(1)	(2)	(3)	(4)	(5)	(6)	(7)	(8)	(9)	(10)	(11)
(1) ΔFC l-PFC—l-MC	1										
(2) ΔFC l-PFC—r-FC	0.73**	1									
(3) ΔFC l-PFC—r-MC	0.77**	0.73**	1								
(4) ΔFC l-MC—r-PFC	0.91**	0.70**	0.73**	1							
(5) ΔFC l-MC—r-MC	0.76**	0.45*	0.77**	0.78**	1						
(6) ΔFC r-PFC—r-MC	0.69**	0.62**	0.92**	0.73**	0.71**	1					
(7) CPM test score	− 0.33	− 0.25	− 0.14	− 0.38	− 0.29	− 0.20	1				
(8) FIQ	− 0.02	0.19	− 0.03	0.01	− 0.11	− 0.01	− 0.11	1			
(9) ACR score	− 0.07	− 0.06	− 0.13	− 0.09	− 0.25	0.02	0.04	0.19	1		
(10) serum BDNF *ng/ml*	0.43*	0.34	0.36*	0.33	0.42*	0.17	− 0.35	− 0.08	− 0.46*	1	
(11) PCS	− 0.11	− 0.15	− 0.10	− 0.13	0.03	− 0.19	0.35	0.4*	− 0.01	− 0.15	1
(12) BDI-II	0.28	0.21	0.13	0.27	0.25	0.08	0.15	0.45*	0.12	0.04	0.45*

BDNF Val/Mel genotype (n = 12)											
	(1)	(2)	(3)	(4)	(5)	(6)	(7)	(8)	(9)	(10)	(11)
(1) ΔFC l-PFC—l-MC	1										
(2) ΔFC l-PFC—r-FC	0.57	1									
(3) ΔFC l-PFC—r-MC	0.66*	0.38	1								
(4) ΔFC l-MC—r-PFC	0.91**	0.77**	0.59*	1							
(5) ΔFC l-MC—r-MC	0.71*	0.22	0.66*	0.57	1						
(6) ΔFC r-PFC—r-MC	0.91**	0.62*	0.69*	0.9**	0.76**	1					
(7) CPM test score	− 0.4	− 0.11	0.08	− 0.55	− 0.21	− 0.48	1				
(8) FIQ	0.16	0.24	0.2	0.36	0.35	0.28	− 0.62	1			
(9) ACR score	0.75**	0.49	0.51	0.82**	0.59*	0.79**	− 0.71*	0.74**	1		
(10) serum BDNF *ng/ml*	0.36	0.5	0.24	0.45	0.17	0.23	0.03	0.14	0.1	1	
(11) PCS	0.21	0.15	0.29	0.33	0.3	0.26	− 0.49	0.69*	0.4	0.47	1
(12) BDI-II	0.21	0.37	− 0.09	0.36	0.06	0.31	− 0.57	0.43	0.4	0.3	0.65*

*BDNF* brain-derived neurotrophic factor*. Val/Val* Val66Val homozygous*. Val/Met* Val66Met heterozygous*. ΔFC* difference in functional connectivity*. l-* left*. r-* right*. PFC* prefrontal cortex*. MC* motor cortex*. CPM* conditioned pain modulation*. FIQ* Fibromyalgia Impact Questionnaire*; ACR score* American College of Rheumatology score for fibromyalgia diagnosis*. PCS* Pain Catastrophizing Scale*. BDI-II* Beck Depression Inventory II.

*p < 0.05.

**p < 0.01.

### Multivariate analysis of the relationship between the ΔFC according to BDNF genotype

**Figure 4:**
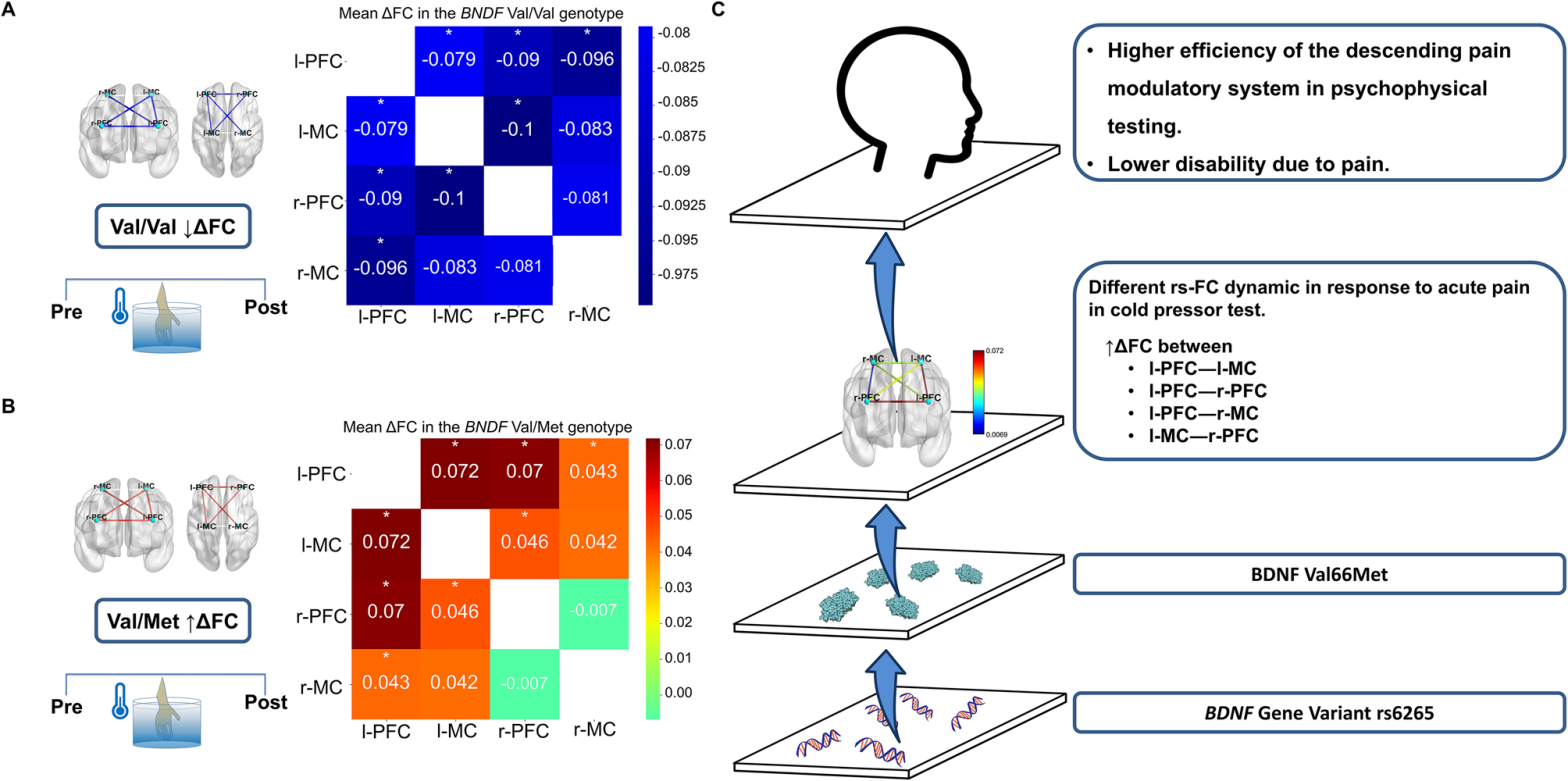
The difference in functional connectivity patterns in response to acute pain according to the *BDNF* genotype and its associations. The Val66Met (rs6265) polymorphism of the *BDNF* gene impacts the changes in FC in response to a cold pressor test in female fibromyalgia patients. Heatmaps presenting the difference in 7 min of resting-state functional connectivity assessed after and before a cold pressor test, in the Val/Val and Val/Met groups. The significant differences between groups of *BDNF* genotypes are presented with an asterisk. (**A**) Data regarding the Val/Val group. The blue edges between regions of interest represent a significant decrease in ΔFC between groups. (**B**) Data regarding the Val/Met group. The red edges represent a significant increase in ΔFC between groups. (**C**) Data associated with the Val/Met group. The Val/Met group presented a significantly ΔFC difference in response to acute pain in the cold pressor test, coupled with lower disability due to pain and performance associated with higher efficiency of the descending pain modulatory system assessed by the conditioned pain modulation test. The colored edges represent ΔFC values as indicated in the heatmap bar. *BDNF* brain-derived neurotrophic factor. *Val/Val* Val66Val homozygous. *Val/Met* Val66Met heterozygous. *ΔFC* functional connectivity delta-value. *rs-FC* resting state functional connectivity. *PFC* prefrontal cortex. *MC* motor cortex. *-l* left. *-r* right. *p < 0.05.

The MANCOVA model using Bonferroni's Multiple Comparison Test revealed a significant relationship between the Val/Met group and the outcomes related to FC (Hotelling's Trace = 1.76, F (6) = 5.47, *p* = 0.001). BDNF genotype Val/Met compared to genotype Val/Val showed higher ΔFC in the following areas: l-PFC—l-MC, l-PFC—r-PFC, l-PFC—r-MC, and l-MC—r-PFC. Regression analysis demonstrated that the serum BDNF was associated with all ΔFC differences between groups, except for the ΔFC between r-PFC—r-MC. In contrast, the ACR score positively correlated with ΔFC in l-MC—r-PFC and r-PFC—r-MC. Results can be seen in Table 4 and Fig. 4.

**Table 4:** MANCOVA analysis of the relationship between the ∆FC regions of interest before and after a cold pressor test according to the *BDNF* Val/Met genotype (n = 42).

Dependent variable	Type III sum of squares	df	Mean square	F	Sig.	Partial eta squared
**Corrected model**						
ΔFC l-PFC—l-MC	0.847^a^	3	0.282	3.853	0.017	0.233
ΔFC l-PFC—r-PFC	0.740^b^	3	0.247	3.787	0.018	0.230
ΔFC l-PFC—r-MC	0.712^c^	3	0.237	3.575	0.023	0.220
ΔFC l-MC—r-PFC	0.906^d^	3	0.302	3.871	0.016	0.234
ΔFC l-MC—r-MC	0.676^e^	3	0.225	2.333	0.089	0.156
ΔFC r-PFC—r-MC	0.683^f^	3	0.228	2.464	0.077	0.163

Parameter	β	SE	t	Sig.	CI 95%	
**Dependent variable: ΔFC l-PFC—l-MC**						
*Intercept*	− 0.622	0.304	− 2.042	0.048	(− 1.23 to − 0.005)	
*BDNF* Val/Val (n = 30)	− 0.257	0.100	− 2.586	0.014	(− 0.45 to − 0.06)	
*BDNF* Val/Met (n = 12)	0^Reference^					
ACR score	0.025	0.012	1.995	0.053	(0.00 to 0.05)	
Serum BDNF (ng/ml)	0.005	0.002	2.708	0.010	(0.001 to 0.01)	
**Dependent variable: ΔFC l-PFC—r-PFC**						
*Intercept*	− 0.445	0.287	− 1.550	0.129	(− 1.02 to 0.14)	
*BDNF* Val/Val (n = 30)	− 0.249	0.094	− 2.656	0.012	(− 0.43 to − 0.06)	
*BDNF* Val/Met (n = 12)	0^Reference^					
ACR score	0.017	0.012	1.451	0.155	(− 0.007 to 0.04)	
Serum BDNF (ng/ml)	0.005	0.002	2.741	0.009	(0.001 to 0.09)	
**Dependent variable: ΔFC l-PFC—r-MC**						
*Intercept*	− 0.442	0.290	− 1.526	0.135	(− 1.03 to 0.14)	
*BDNF* Val/Val (n = 30)	− 0.226	0.095	− 2.390	0.022	(− 0.42 to − 0.04)	
*BDNF* Val/Met (n = 12)	0^Reference^					
ACR score	0.015	0.012	1.298	0.202	(− 0.009 to 0.04)	
Serum BDNF (ng/ml)	0.005	0.002	2.829	0.007	(0.002 to 0.009)	
**Dependent variable: ΔFC l-MC—r-PFC**						
*Intercept*	− 0.731	0.314	− 2.327	0.025	(− 1.37 to − 0.09)	
*BDNF* Val/Val (n = 30)	− 0.260	0.103	− 2.533	0.016	(− 0.47 to − 0.05)	
*BDNF* Val/Met (n = 12)	0^Reference^					
ACR score	0.029	0.013	2.227	0.032	(0.003 to 0.06)	
Serum BDNF level (ng/ml)	0.006	0.002	2.643	0.012	(0.001 to 0.01)	
**Dependent variable: ΔFC l-MC—r-MC**						
*Intercept*	− 0.490	0.349	− 1.404	0.169	(− 1.19 to 0.21)	
*BDNF* Val/Val (n = 30)	− 0.216	0.114	− 1.893	0.066	(− 0.45 to 0.02)	
*BDNF* Val/Met (n = 12)	0^Reference^					
ACR score	0.018	0.014	1.239	0.223	(− 0.011 to 0.05)	
Serum BDNF (ng/ml)	0.005	0.002	2.293	0.027	(0.001 to − 0.01)	
**Dependent variable: ΔFC r-PFC r-MC**						
*Intercept*	− 0.808	0.342	− 2.364	0.023	(− 1.50 to − 0.12)	
*BDNF* Val/Val (n = 30)	− 0.183	0.112	− 1.633	0.111	(− 0.40 to 0.04)	
*BDNF* Val/Met (n = 12)	0^Reference^					
ACR score	0.031	0.014	2.209	0.033	(0.003 to 0.06)	
Serum BDNF (ng/ml)	0.005	0.002	2.006	0.052	(− 4.09 to 0.009)	

*Df* degrees of freedom*. χ2* Wald Chi-Square*. CI* confidence interval*. β* regression coefficient*. SE* standard error*. Sig. p*-value. *BDNF* brain-derived neurotrophic factor*. ACR* American College of Rheumatology*. ΔFC* functional connectivity*. Val/Val* Val66Val homozygous*. Val/Met* Val66Met heterozygous*. PFC* prefrontal cortex*. MC* motor cortex*. -l* left*. -r* right*. ACR score* American College of Rheumatology score for fibromyalgia diagnosis*. BDI-II* Beck Depression Inventory II.

^a^R Squared = 0.233 (Adjusted R Squared = 0.173).

^b^R Squared = 0.230 (Adjusted R Squared = 0.169).

^c^R Squared = 0.220 (Adjusted R Squared = 0.159).

^d^R Squared = 0.234 (Adjusted R Squared = 0.174).

^e^R Squared = 0.156 (Adjusted R Squared = 0.089).

^f^R Squared = 0.163 (Adjusted R Squared = 0.097).

### Secondary outcomes: multivariate analysis to examine the efficiency of DPMS, time of pain reactivity, and quality of life due to fibromyalgia symptoms according to genotypes

A generalized linear mixed model (GLMM) revealed a main effect according to the BDNF genotype in both DPMS evaluated by the change in the NPS (0–10) and quality of life assessed by the FIQ. The heterozygotes showed lower dysfunction on the DPMS. Despite the genotypes, the function of DPMS was negatively correlated with serum BDNF and ACR scores. In contrast, a higher score on the FIQ was positively correlated with a higher ACR score. Lower quality of life was directly related to the severity of ACR, a composite index by sums of the WPI index and SSS. Data are presented in Table 5.

**Table 5:** Generalized linear model analysis of the relationship between the *BDNF* Val66Met genotype, serum BDNF and ACR score according to DPMS efficiency and quality of life due to fibromyalgia symptoms (n = 42).

	β	SE	CI 95%	Wald χ2	df	Sig
**Dependent variable: Change on Numerical Pain Scale (NPS0-10) during conditioned pain modulation test**						
*Intercept*	2.103	1.9808	(− 1.780 to 5.985)	1.127	1	0.288
*BDNF* Val/Val (n = 30)	1.419	0.6931	(0.061 to 2.778)	4.191	1	0.041
*BDNF* Val/Met (n = 12)	0^Reference^					
Serum BDNF (ng/ml)	− 0.028	0.0132	(− 0.054 to 0.002)	4.393	1	0.036
ACR score	− 0.155	0.0811	(− 0.314 to 0.004)	3.666	1	0.056
**Dependent variable: Fibromyalgia Impact Questionnaire**						
*Intercept*	6.635	15.5828	(-23.907 to 37.177)	0.181	1	0.670
*BDNF* Val/Val (n = 30)	6.895	5.2733	(-3.440 to 17.231)	1.710	1	0.191
*BDNF* Val/Met (n = 12)	0^Reference^					
Serum BDNF (ng/ml)	0.084	0.1035	(-0.119 to 0.287)	0.656	1	0.418
ACR score	2.325	0.6415	(1.068 to 3.582)	13.135	1	0.0003

*Df* degrees of freedom*. χ2* Wald Chi-Square*. CI* confidence interval*. β* regression coefficient*. SE* standard error*. Sig. p*-value. *BDNF* brain-derived neurotrophic factor*. ACR* American College of Rheumatology *Val/Val* Val66Val homozygous*. Val/Met* Val66Met heterozygous.

The Cramer’s V was used as a measure of effect size for qui-square tests. The size effect was interpreted as follows: Standards for interpreting Cramer’s V as proposed by Cohen (1988) are the following: DF (degrees of freedom) = 1 (0.10 = small effect) (0.30 = medium effect) (0.50 = large effect). https://www.campbellcollaboration.org/escalc/html/EffectSizeCalculator-R5.php.

## Discussion

These findings reveal that the Val/Met group is associated with an increased ΔFC between the PFC and MC. In contrast, the Val/Val group showed a decreased interhemispheric connectivity in these areas, less active engagement of DPMS, and a higher impact of fibromyalgia symptoms on quality of life. Furthermore, we found that despite genotypes, the serum BDNF and scores on ACR are positively correlated to ΔFC in the PFC and MC areas. These results highlight that FM patients carrying the *BDNF* Val/Met polymorphism might be less prone to maladaptive neuroplasticity, as indicated by the higher efficiency of DPMS and less severe fibromyalgia symptoms. However, since the effects of *BDNF* in the nervous system are complex, it is possible that specific compensatory mechanisms, in terms of brain plasticity, may occur at least partially due to genetic differences^43^. Therefore, a single explanation for our findings is too ambitious, and parsimony is required to translate them into the clinical setting.

Our findings may be explained by the hypothesis that the increase in ΔFC associated with the *BDNF* Val66Met reflects a cortical response to pain in response to painful stimuli. Since earlier studies have found that the *BDNF* Val/Met polymorphism may be associated with the modulation of neuroplasticity^44–46^, the phenomenon described in the current study may be related to differences in neuroplasticity between both groups due to the *BDNF* function. Furthermore, Val/Met genotype was associated with a distinct propensity to FM symptoms, which seems to indicate that *BDNF* also could modulate the emergence of the FM phenotype. This hypothesis is corroborated by a growing body of evidence that subjects with *BDNF* Val66Met polymorphism display differences in brain plasticity expressions as induced by motor learning^46^, brain stimulation^44^, and experimental pain stimulation^47^. In line with the present results, it is reasonable to ask if *BDNF* Val66Met polymorphism may contribute to differences in the effect of neuromodulation therapeutic and behavior related to pain. Aligned with this perspective, the positive ΔFC in response to CPT expressed by Val/Met FM patients indicates that this genotype is significant for the PFC—MC activation map.

Remarkably, the current findings’ relevance lies in the fact that the *BDNF* Val66Met genotype significantly discriminates between two ΔFC patterns in response to acute pain stimulus. Thus, our findings give input on the criticality of this polymorphism as a differential factor for the functional fingerprint of acute pain response in female FM patients. This may explain the influence of BDNF on the degree and quality of response to treatment in various chronic pain conditions (e.g., tDCS, electroacupuncture, among others)^48–51^. The association between serum BDNF levels and the Val66Met polymorphism with DPMS function may be either due to an independent effect of both factors or because BDNF serum levels are linked with the respective polymorphism, which is plausible when considering that the Val66Met leads to changes in concentration of serum BDNF in some conditions^52^.

Moreover, the results indicate that serum BDNF was associated with increased FC in response to noxious stimulus across all but one of the ROI-ROIs analyzed, nominally ΔFC r-PFC—r-MC. This is paramount for representing a consistent direction towards which BDNF protein seems to influence changes in MC—PFC FC following acute pain, that is, as a positive function of its serum levels. The ACR score was positively associated with ΔFC in l-MC—r-PFC and ΔFC in r-PFC—r-MC, implying a higher widespread pain and symptom severity associated with an increase in this ROI-ROIs FC as a response to noxious stimulation. ACR was not associated with ΔFC in other ROI-ROIs but was unsurprisingly associated with lower quality of life (i.e., positive association with FIQ). Val/Val genotype had been previously associated with higher pain catastrophizing in FM^53^.

Consequently, this intricate pattern prevents the present study from putatively defining one of the genotypes (i.e., Val/Met or Val/Val) as related to more severe symptoms, with more studies being required to address this subject. For now, this polymorphism seems to have some impact on the functional response to pain as assessed by ΔFC across MC and PFC. Perhaps the effect that this differential pattern has on the global clinical status of FM patients will not be shown to be more than subtle. However, it might have value as a biomarker for follow-up and or response to treatment.

In this sense, it is remarkable that an SNP in a critical gene for neuroplasticity modulation is sufficient to cause different brain responses to acute noxious stimuli in patients with a chronic pain disease. Nevertheless, the influence of multiple genetic variants on brain states needs to be further explored. Further studies should investigate if these polymorphism-dependent differences in the functional fingerprint of FM patients are dynamically set throughout development by a continuous modulation of experience-dependent neuroplasticity^46^ or if they represent a consistent pattern since birth owing predominantly to genetic factors.

Although these results suggest an association between pain processing and brain functional signatures among the *BDNF* Val66Met polymorphism genotypes, several limitations of the present study must be considered. First, this is an exploratory analysis with a few participants compared to other neuroimaging genetics surveys. Second, this study compared functional connectivity, psychophysical, and clinical parameters between subgroups of patients with fibromyalgia. This is within the scope of characterizing clusters of a prototypical primary chronic pain disease by the severity of symptoms and looking for potential phenomena-related underlying mechanisms. This approach has as a limitation the absence of comparisons with healthy controls and, therefore, no extrapolation of these findings compared to the healthy population. Third, the effects of the *BDNF* Val66Met polymorphism on the noxious-evoked ΔFC between the MC and the PFC was our primary interest due to the critical role these ROIs have in NIBS, resulting in a montage focused on these cortical areas. Furthermore, although the optode positioning matches more specifically the DLPFC than other PFC regions, we chose to refer to this ROI more generically as PFC since fNIRS do not present a spatial resolution as high as other neuroimaging methods and since we did not utilize any neuronavigation device. Fourth, only Val/Val and Val/Met were included due to our population's low prevalence of individuals with the Met/Met genotype^54^. Fifth, only right-handed women were included in this study, hindering the assessment of results differentially associated with lateralization of brain function and the extrapolation of these results to male FM patients. Finally, this is a cross-sectional study; further research with larger sample sizes in longitudinal studies is needed to evaluate the dynamics of the Val66Met effects in FM, such as in response to treatment and symptom evolution.

These results indicate that the FM patients carrying the Val/Met genotype are prone to show increased FC across MC and PFC response to a standardized acute nociceptive stimulus. Besides, Val/Met genotype is related to the higher efficiency of DPMS and less severe fibromyalgia symptoms. These findings suggest that this genetic factor might contribute to individual differences when experiencing pain and perhaps affect the vulnerability to the emergence of chronic pain syndromes.

## Data Availability

The datasets used and/or analyzed during the current study are available from the corresponding author on reasonable request.
